# The Mississippi Valley Medical Association

**Published:** 1898-08

**Authors:** 


### THE MISSISSIPPI VALLEY MEDICAL ASSOCIATION.

The twenty-fourth annual meeting of the Mississippi Valley Medical Association will be held at Nashville, Tenn., October 11-14, under the presidency of Dr. John Young Brown, of St. Louis, Mo.

This association is second in size only to the American Medical Association and has done most excellent scientific work in the past. The annual address will be made by Dr. James T. Whittaker, of Cincinnati, on Medicine, and by Dr. Ben Johnson, of Richmond, Va., on Surgery. The mere mention of the names of these gentlemen establishes the fact that the Association will hear two scholarly and scientific addresses.

Nashville is a most excellent convention city and is well equipped with hotels, and with the record of the meeting in Louisville in 1897 as an example, the local profession, under the leadership of Dr. Duncan Eve as Chairman of the Committee of Arrangements has prepared to have a better meeting.

Already titles of papers are being received. These should be sent to the secretary, Dr. Henry E. Tuley, No. 111 West Kentucky Street, Louisville, Ky., as early as possible to insure a good place upon the program. Reduced rates on all railroads will be granted on the certificate plan.

